# Web-Based Application Based on Human-in-the-Loop Deep Learning for Deidentifying Free-Text Data in Electronic Medical Records: Development and Usability Study

**DOI:** 10.2196/46322

**Published:** 2023-08-25

**Authors:** Leibo Liu, Oscar Perez-Concha, Anthony Nguyen, Vicki Bennett, Victoria Blake, Blanca Gallego, Louisa Jorm

**Affiliations:** 1 Centre for Big Data Research in Health University of New South Wales Sydney Australia; 2 Australian e-Health Research Centre (AEHRC) Commonwealth Scientific and Industrial Research Organisation (CSIRO) Brisbane Australia; 3 Metadata, Information Management and Classifications Unit (MIMCU) Australian Institute of Health and Welfare Canberra Australia; 4 Eastern Heart Clinic Prince of Wales Hospital Randwick Australia

**Keywords:** web-based system, deidentification, electronic medical records, deep learning, narrative free text, human in the loop, free text, unstructured data, electronic health records, machine learning

## Abstract

**Background:**

The narrative free-text data in electronic medical records (EMRs) contain valuable clinical information for analysis and research to inform better patient care. However, the release of free text for secondary use is hindered by concerns surrounding personally identifiable information (PII), as protecting individuals' privacy is paramount. Therefore, it is necessary to deidentify free text to remove PII. Manual deidentification is a time-consuming and labor-intensive process. Numerous automated deidentification approaches and systems have been attempted to overcome this challenge over the past decade.

**Objective:**

We sought to develop an accurate, web-based system deidentifying free text (DEFT), which can be readily and easily adopted in real-world settings for deidentification of free text in EMRs. The system has several key features including a simple and task-focused web user interface, customized PII types, use of a state-of-the-art deep learning model for tagging PII from free text, preannotation by an interactive learning loop, rapid manual annotation with autosave, support for project management and team collaboration, user access control, and central data storage.

**Methods:**

DEFT comprises frontend and backend modules and communicates with central data storage through a filesystem path access. The frontend web user interface provides end users with a user-friendly workspace for managing and annotating free text. The backend module processes the requests from the frontend and performs relevant persistence operations. DEFT manages the deidentification workflow as a project, which can contain one or more data sets. Customized PII types and user access control can also be configured. The deep learning model is based on a Bidirectional Long Short-Term Memory-Conditional Random Field (BiLSTM-CRF) with RoBERTa as the word embedding layer. The interactive learning loop is further integrated into DEFT to speed up the deidentification process and increase its performance over time.

**Results:**

DEFT has many advantages over existing deidentification systems in terms of its support for project management, user access control, data management, and an interactive learning process. Experimental results from DEFT on the 2014 i2b2 data set obtained the highest performance compared to 5 benchmark models in terms of microaverage strict entity–level recall and *F*_1_-scores of 0.9563 and 0.9627, respectively. In a real-world use case of deidentifying clinical notes, extracted from 1 referral hospital in Sydney, New South Wales, Australia, DEFT achieved a high microaverage strict entity–level *F*_1_-score of 0.9507 on a corpus of 600 annotated clinical notes. Moreover, the manual annotation process with preannotation demonstrated a 43% increase in work efficiency compared to the process without preannotation.

**Conclusions:**

DEFT is designed for health domain researchers and data custodians to easily deidentify free text in EMRs. DEFT supports an interactive learning loop and end users with minimal technical knowledge can perform the deidentification work with only a shallow learning curve.

## Introduction

Narrative free-text data in electronic medical records (EMRs) include a variety of clinical documents such as consultation notes, nursing notes, progress notes, and discharge summaries, which contain valuable information for analysis and research to inform better patient care [1-3]. The free-text data can include personally identifiable information (PII), for example, patient name, date of birth, address, phone number, and patient identifier, which can be used to identify an individual on its own or with other information. It is necessary to deidentify the free-text data by removing this PII before releasing to researchers for secondary purposes where the reuse of the data is not covered in patients’ informed consent forms or when requested as part of a waiver of informed consent by an institutional review board or any other human research ethics committees, as required by legislation including the Privacy Rule of the HIPAA (Health Insurance Portability and Accountability Act) [4] in the United States and the Privacy Act, 1988 [5], in Australia. However, manual deidentification has been proven to be a time-consuming and labor-intensive task [6].

In the past decade, researchers have investigated many different automated deidentification approaches including rule-based matching [7-9] and machine learning (ML) models [10-14]. Hand-written regular expressions and ad hoc knowledge dictionaries are used in rule-based deidentification approaches for a specific free-text data set [15]. In contrast, ML-based deidentification approaches use manually annotated data to train named entity recognition (NER) models, enabling the prediction of PII entities from free-text data. Although rule-based methods do not necessitate the preparation of annotated data, the rules are challenging to generalize to other corpora without manual adjustments from experienced domain experts [16]. The Bidirectional Long Short-Term Memory-Conditional Random Field (BiLSTM-CRF) has been proven to achieve state-of-the-art or competitive results on the free text deidentification task [17-19]. With the success of transformer models in the NLP domain, some studies began to explore its use on deidentification tasks [20-22]. Johnson et al [21] fine-tuned pretrained transformer models and achieved a binary token-level *F*_1_-score of 0.984 on the 2014 i2b2 test set. However, a benchmark study [20] showed that BiLSTM-CRF achieved better performances compared to transformer-based models. Another study conducted by Tang et al [17] demonstrated that incorporating the pretrained transformer language model as the word embedding layer in BiLSTM-CRF led to an improvement in the *F*_1_-scores on the 2014 i2b2 deidentification data set, compared to using other word embeddings (eg, Word2Vec and ELMo). Furthermore, several ensemble approaches that combine multiple individual ML models have been proposed on deidentification tasks [14,23,24]. By leveraging the strengths of individual methods, these ensemble methods have demonstrated improved performance on deidentification tasks.

The traditional workflow of the ML-based deidentification approaches consist of three stages: (1) annotation: human annotators manually tag all the PII in the free text. The interannotator agreement is calculated to measure the quality of the annotation [13]; (2) model training: ML experts train models using the annotated free text; and (3) deidentification: the PII predicted by the models are substituted by surrogates or tags or removed completely. Although pretrained ML solutions can potentially be used “out of the box,” there are significant variations between hospitals, vendors, and countries in the structure and content of EMRs and the nature of PIIs. Furthermore, data custodians may require performance metrics based on their specific data before gaining sufficient confidence to use these tools. Therefore, manual annotation remains a time-consuming process and is the main bottleneck in training ML-based deidentification models [25,26]. To overcome this, several annotation tools (eg, BRAT [27] and WAT [14]) have been used to speed up the annotation stage [6,28]. Nevertheless, the second and third stages of the workflow still require considerable input from ML experts. In recent years, some annotation tools (eg, ezTag [29], INCEpTION [30], and Prodigy [31]) have integrated interactive learning for iteratively retraining the models using the latest annotated free text to provide preannotation suggestions, which automates the second stage of the workflow. These tools can be used to handle some of the deidentification task (ie, PII tagging). Aberdeen et al [32] developed an open-source deidentification tool, MITRE Identification Scrubber Toolkit (MIST), which comprises a web-based graphical annotation tool, a training module, a tagging module, a redaction and resynthesis module, and an experiment engine. These modules work together using a “tag-a-little, learn-a-little” loop strategy to complete the deidentification task, bypassing the need for ML experts for the second and third stages of the traditional deidentification workflow. The annotation tool is used by annotators to tag the PII from the free-text files. The training module trains a conditional random field-based sequence tagger using these annotated files. The tagging module automatically tags the PII for the new files that can be manually corrected by the annotators. Furthermore, MIST provides a workspace mode to conveniently manage a corpus which needs to be deidentified. However, many operations of MIST need to be done via the command line, for example, creating a workspace, importing files into a workspace, and training models. The MIST server needs to be restarted to get the newly trained model into effect. Moreover, the end users of the MIST tool are required to have the technical knowledge to run command lines.

Off-the-shelf tools [33] such as Amazon Comprehend Medical [34], Clinacuity CliniDeID [35], and National Library of Medicine (NLM) Scrubber [36], can be used to deidentify free text directly without following the traditional deidentification workflow. These 3 tools are all HIPAA-compliant, following the HIPAA’s “Safe Harbor” method [14] to remove 18 types of identifiers. Amazon Comprehend Medical is a cloud-based service which needs the data be uploaded to its service end point. This represents a significant barrier to adopting it for use with EMRs, which are stored in a secure and internet access-restricted environment. CliniDeID, originally a commercial clinical text deidentification software, has recently been made available as free open-source software since November 2022. This allows for additional retraining on specific data sets to enhance the model's performance. Although NLM Scrubber can be installed locally, its performance cannot be improved because it has no ability to learn from the end users’ free-text data, which may vary considerably from the data used to develop the tools. Therefore, data custodians are responsible for reviewing and evaluating the deidentification results provided by these off-the-shelf tools to ensure it meets their benchmarks. Another main obstacle for adopting the off-the-shelf tools is that the PII types present in specific free-text data outside of a HIPAA covered entity or country (such as Australia) can be different from the 18 HIPAA PII types.

In this study, we designed and implemented a web-based system, deidentifying free text (DEFT), for tagging and substituting designated PII in free-text data in EMRs with human (annotators) in the loop. The system can be readily and easily adopted for the free text deidentification task in secure and internet access–restricted network environments. The main features of the system are listed below:

- Web-based: the web-based deidentification system can be easily accessed by multiple end users via a web browser. Compared to desktop-based applications, web-based applications are less hardware dependent and can easily be updated and upgraded.
- Powered by deep learning models: we used a deep learning NER model to recognize and tag PII entities from free-text data.
- Preannotation bootstrapped by a semiautomatic learning loop: following the learning loop we have proposed previously [14], DEFT preannotates the free text using the ML model that is automatically trained on the previous annotated free text completed by the annotators.
- Customized PII types: the PII types can vary depending on the free text corpus and different data sharing scenarios. For example, full dates may need to be kept for future research in a secure environment which can only be accessed by ethically approved users [14].
- Suitable for nontechnical end users: experienced health-domain annotators can complete the whole deidentification task using DEFT. No technical or ML knowledge is required.
- Simple and task-focused web user interface (UI): DEFT makes the annotators focus on the annotation work through a simple and well-designed web UI.
- Implements autosave: each annotation action, including annotator name, PII entity positions, PII type, and annotation time, is saved automatically.
- Fewer clicks for annotation: fewer clicks mean quick annotation and less deidentification time.
- Supports project management and team collaboration: the deidentification task can be managed as a project, which can contain one or more data sets. The team annotators can work on the same data set in the project at the same time to accelerate the annotation process.
- Implements user access control: only approved users can access specific projects.
- Uses central data storage: all the data can be stored in 1 central location which the DEFT server can access through filesystem path. This avoids importing or transferring thousands or millions of small free-text files across the network.

## Methods

### System Architecture

**Figure 1:**
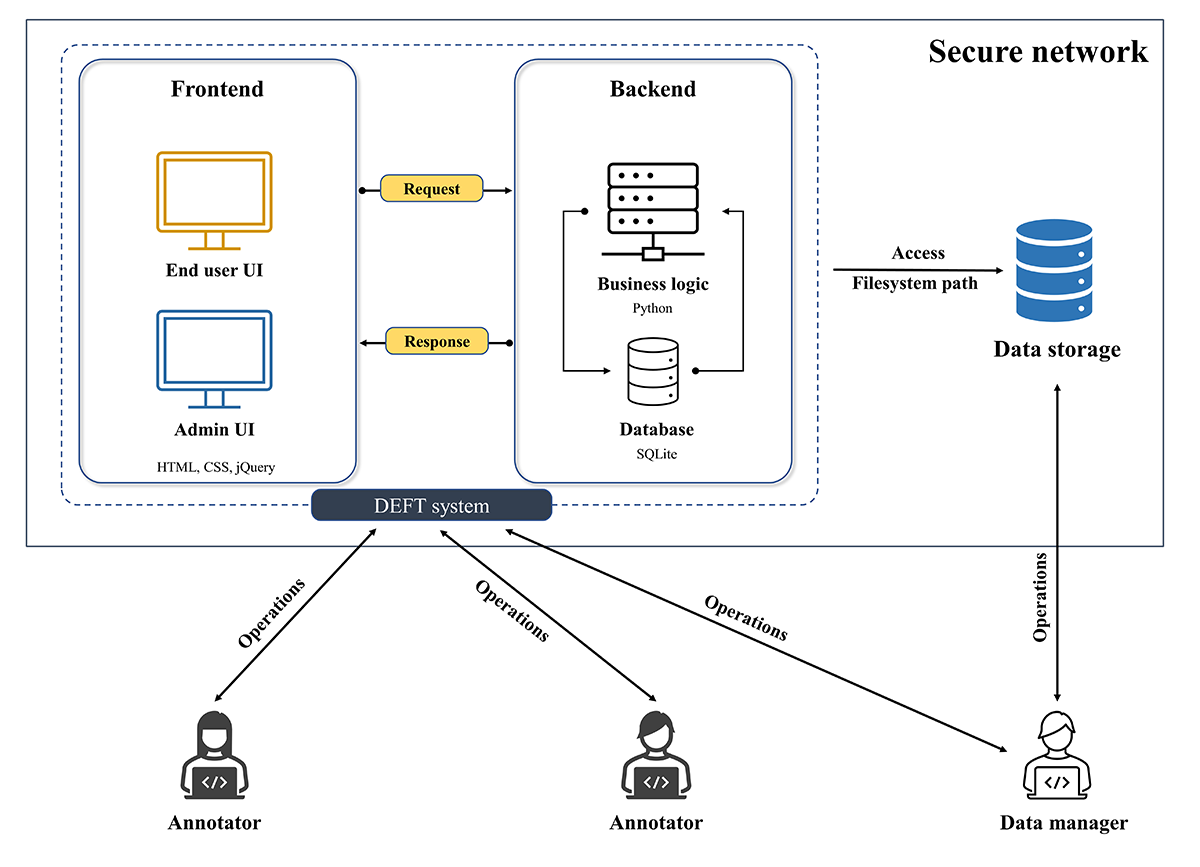
Overview of the DEFT system architecture. DEFT: deidentifying free text; UI: user interface.

DEFT has been designed to be simple and manageable, so end users can easily conduct the deidentification work on their own free-text data that are stored in a secure and internet access–restricted environment. Figure 1 shows the overview of the DEFT system architecture. DEFT communicates with the central data storage by using a filesystem path to retrieve the free-text data and generate deidentified data. The annotators remotely access the DEFT web UI to annotate the free text via their own devices. DEFT is responsible for all the business logic processing. The data manager helps to manage projects and data through the DEFT web UI and direct connection to the data storage, respectively.

We implemented DEFT using Django, a high-level Python web framework. As shown in Figure 1, DEFT comprises frontend and backend modules. The frontend module was built with HTML, CSS, and jQuery and has 2 different web UIs including an end user UI and admin UI. The former is used by the annotator to tag the PII and the latter is for the data manager to manage users, models, projects, and PII types. The backend consists of 2 components, that is, business logic and database. The first one is the controller that receives the requests from the frontend and invokes the relevant business logic to produce the responses which are sent back to the frontend. The second one has the persistence component built with SQLite and interacts with the business logic component to store all the application data such as project information, and PII positions and types.

### Project-Based Deidentification Work

**Figure 2:**
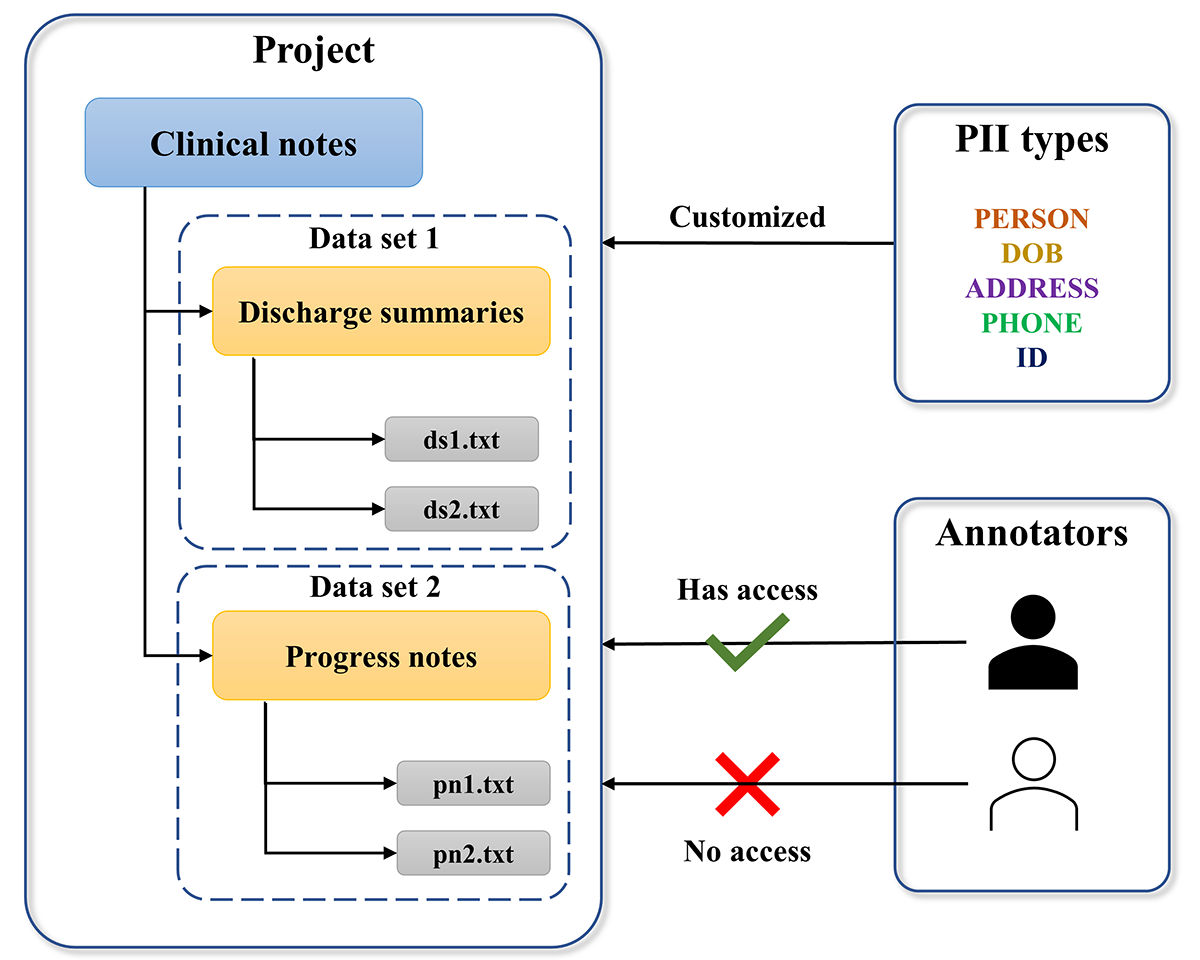
An example of a deidentification project structure and access control. DOB: date of birth; PII: personally identifiable information.

In the DEFT system, the deidentification work is organized as projects which must have at least 1 data set. Multiple projects can be processed at the same time. A project is created by the users for a deidentification task of a new free text corpus. Multiple data sets can be added into the project according to the users’ requirements. The users can flexibly customize any number of PII types with different display colors for the project and control who has access to the project. The system does not provide default PII types, because these can vary depending on the project. The raw data files are stored in specific data storage outside the DEFT system and the access path is configured in DEFT for the relevant project. Users can import the data files (txt format) into DEFT manually or wait for the system to import them automatically. The importing operation only saves the file names into the DEFT database rather than the file contents so that all the identified data can be safely maintained in specific data storage. Figure 2 shows an example of a deidentification project structure. A project named “Clinical Notes” is created and 2 data sets are added with the names “Discharge Summaries” and “Progress Notes,” respectively. A list of PII types (PERSON, date of birth [DOB], ADDRESS, PHONE, ID) are configured for the project according to the users’ requirements. The user shown in solid black is assigned access to the project as the annotator. All the project configurations can be done via the admin UI by the data managers of the team.

### Deep Learning NER Model

**Figure 3:**
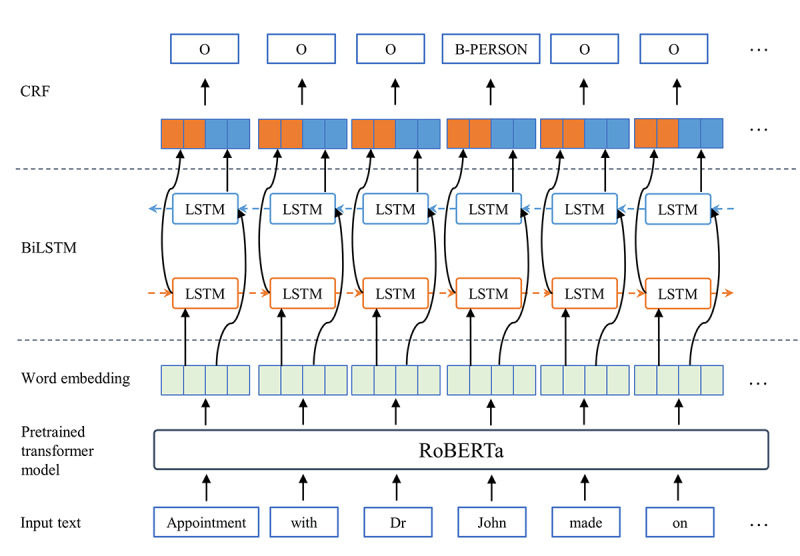
Deep learning named entity recognition model architecture. BiLSTM: Bidirectional Long Short-Term Memory; CRF: conditional random field; LSTM: Long Short-Term Memory.

In the DEFT system, we used the BiLSTM-CRF architecture to train a NER model that can identify word spans related to specific PII types in the working text. Although an ensemble model may perform better than a single BiLSTM-CRF model, we selected the latter for DEFT considering its competitive performance and faster training time, noting that DEFT may be deployed in settings where computing resources are constrained. The Python library FLAIR [37] was used to implement the BiLSTM-CRF NER model. The pretrained RoBERTa model [38] was selected to generate input representations in our model. Figure 3 shows the model architecture.

### Learning Loop

**Figure 4:**
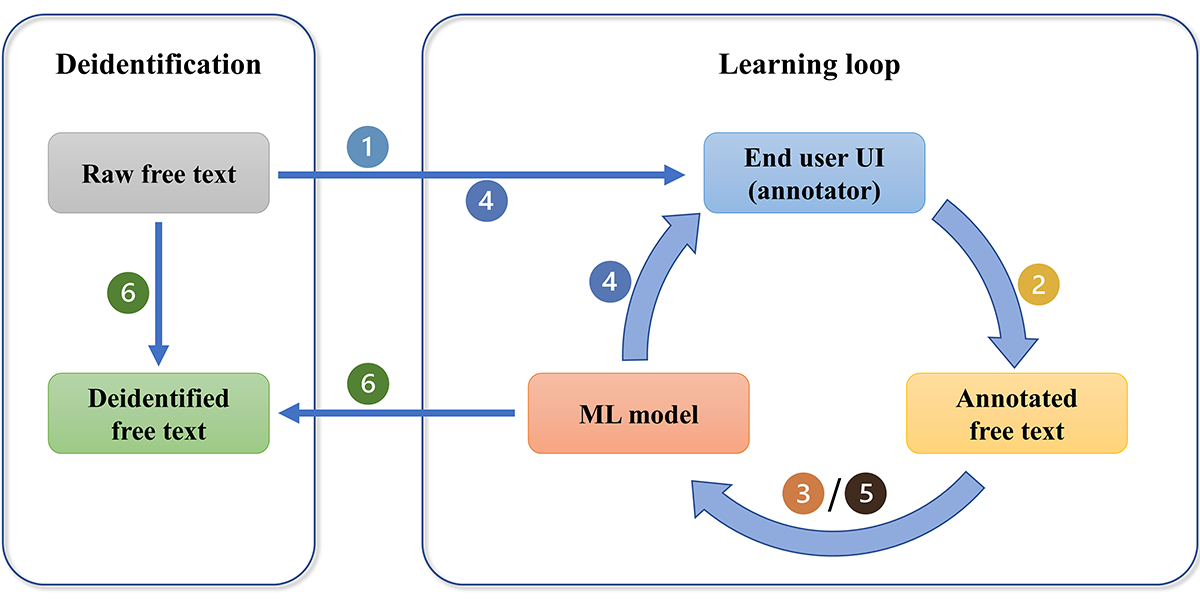
Learning loop of DEFT system. (1) Feed raw free text into DEFT system; (2) annotate raw free text or correct preannotated free text; (3) train ML models; (4) preannotate raw free text; (5) iterative ML model training; and (6) deidentify raw free text. DEFT: deidentifying free text; ML: machine learning; UI: user interface.

**Figure 5:**
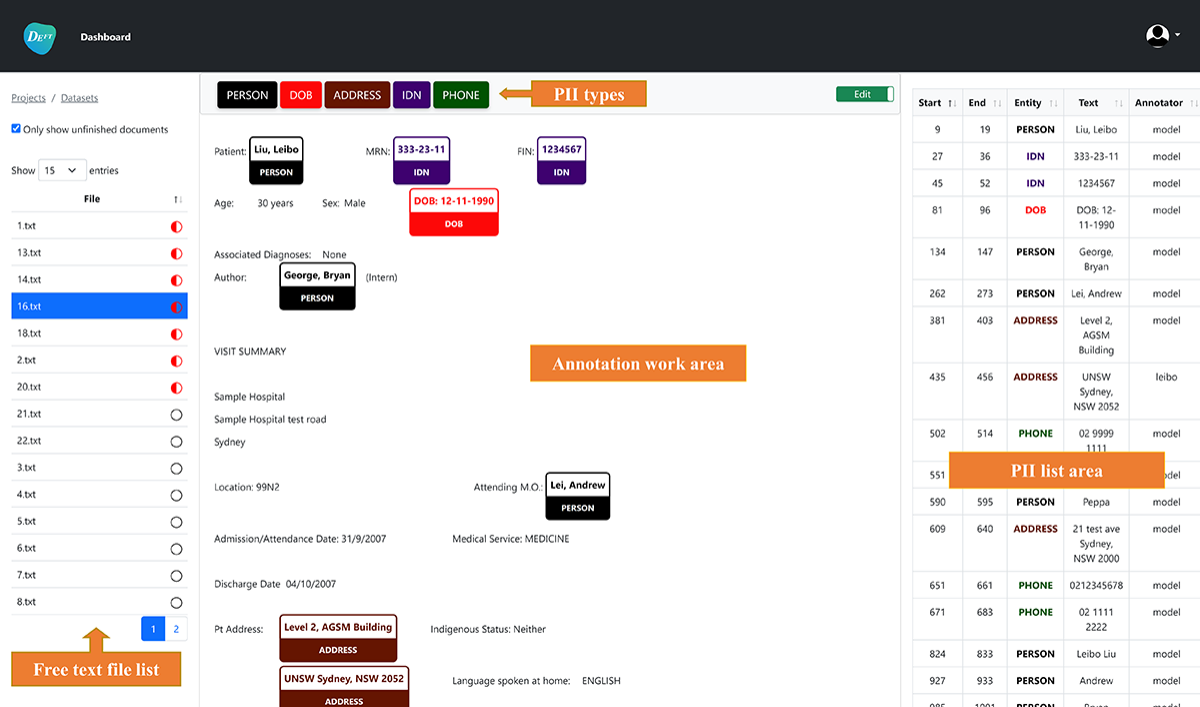
A demo of the end user UI for the annotators. The patient information is dummy data. PII: personally identifiable information; UI: user interface.

The core of the DEFT system’s workflow is a learning loop, which comprises 3 elements: end user UI, annotated free text, and ML model, as shown in Figure 4. At the beginning of the deidentification work for a new project, there is no ML model in the system. The deidentification process follows the following six steps:

1. Feed raw free text into DEFT system. An initial set of raw free-text files are loaded into the end user UI where the annotators begin to manually tag PII in the raw free text. The end user UI provides a simple and task-focused interface to the annotators so that the annotation can be done quickly and easily.
2. Annotate raw free text or correct preannotated free text. Figure 5 shows a screenshot of the end user UI which contains 4 main areas: file list area, PII type area, annotation work area, and PII list area. First, the annotator selects 1 file in the file list area to load its free text content into the annotation work area and clicks on 1 PII type in the PII type area. Second, the annotator reviews the free text and tags the words using double left click or word spans using click drag-and-release that are related to the selected PII type. The PII entities will be surrounded by colored boxes with the PII type underneath the words. Incorrectly tagged PII entities can be removed by single left click on the entity text. Finally, the tagged PII entities are listed in the PII list area with detailed information about start index, end index, PII entity type, PII entity text, and annotator name. The annotator needs to change the “Edit” mode to “Complete” mode for marking the completion of the annotation work for the current working file. The above mentioned PII entity details, except PII entity text, are automatically saved in the DEFT database so that they can be integrated with the raw free-text file contents to generate the annotated free-text files for model training. Furthermore, not having PII entity text stored in the DEFT system protects the identified data.
3. Train ML models. Once the completed amount of the annotated free-text files reaches the preconfigured model retraining threshold which is the number of clinical notes (eg, 200 clinical notes), the system starts the ML model training process, which splits the annotated data into training, validation, and test sets. The model is automatically trained in the backend and loaded into the system after that.
4. Preannotate raw free text. Another small set of free-text files, that has been preannotated by the trained model from Step 3, are assigned to the annotators to add the PIIs which the model does not preannotate or to correct incorrect PIIs that the model has preannotated, via the end user UI. The added or corrected PII entity details are added into the system database.
5. Iterative ML model training. If the number of the new completed files reaches the retraining threshold, it will trigger the ML model to be retrained on all the annotated free-text files. Steps 2, 3, and 4 are iteratively conducted until the performance of the model meets the specified benchmark for the data set. In the DEFT system, we use the strict entity-level microaverage *F*_1_-score, which is the primary metric in previous deidentification challenges [16,39,40], to evaluate the performance of the model and deidentification work.
6. Deidentify raw free text. The final model is used to deidentify the remaining free text in the data set, including automatically tagging the PIIs and replacing the PIIs with the special tags. For example, the person names are replaced by “<**PERSON**>” in the deidentified free text.

### DEFT Functionalities

Table 1 describes the main functionalities of the DEFT system which are grouped according to the 2 DEFT web UIs. The admin UI includes the management components of the key elements such as projects, data sets, PII types, and users. The functionalities of the end user UI are mainly focused on the annotation work, for example, tagging the PII, changing the file status, and preannotation. Currently, only txt format data files are supported in DEFT. There are two export options: (1) export the annotated data files in the XML format which include the raw free text and all the PII entities (Figure S1 in Multimedia Appendix 1); the annotated XML data files could be a valuable data source for future deidentification research; and (2) export the deidentified data files in TXT format (Figure S2 in Multimedia Appendix 1). The file contents are the same as the original one except that the PII words are replaced by the special tags.

**Table 1:** The main functionalities of the DEFT^a^ system.

UI^b^ and functionality		Description		
**Admin**				
	Project management	Create or delete or modify project information Configure the project data path Assign user access right		
	Data set management	Create or delete or modify data set information Import data file names Export all the annotated data files Export all the deidentified data files		
	Data file management	Create or delete or modify data files Export single annotated data file Export single deidentified data file		
	PII^c^ type management	Create or delete or modify PII types		
	Model management	Review all the trained models of the project		
	User management	Create or delete or modify users		
**End user**				
	Project list	List accessible projects		
	Data set list	List the data sets of the selected projects		
	Annotation dashboard	List all the files of the selected data set Open 1 file Tag the PII entities in the free text		
	Auto save	The add or remove PII actions are saved automatically		
	Preannotation	Pretag the possible PII entities by the trained ML^d^ model when the users open a file		
	Hide completed files	Filter completed files out from the data file list		
	File status management	Switch the file status between “Edit” and “Complete”		

^a^DEFT: deidentifying free text.

^b^UI: user interface.

^c^PII: personally identifiable information.

^d^ML: machine learning.

### Ethics Approval

This study has obtained ethical approval from the South Eastern Sydney Local Health District Human Research Ethics Committee (reference 2019/ETH12625) and the Population Health Services Research Ethics Committee (reference number 2020/ETH01614). The ethics committees allow the data usage for this study without additional consent. The experiments in this study were conducted in the E-Research Institutional Cloud Architecture [41], a secure cloud computing infrastructure for individuals working with sensitive data.

## Results

### Overview

**Figure 6:**
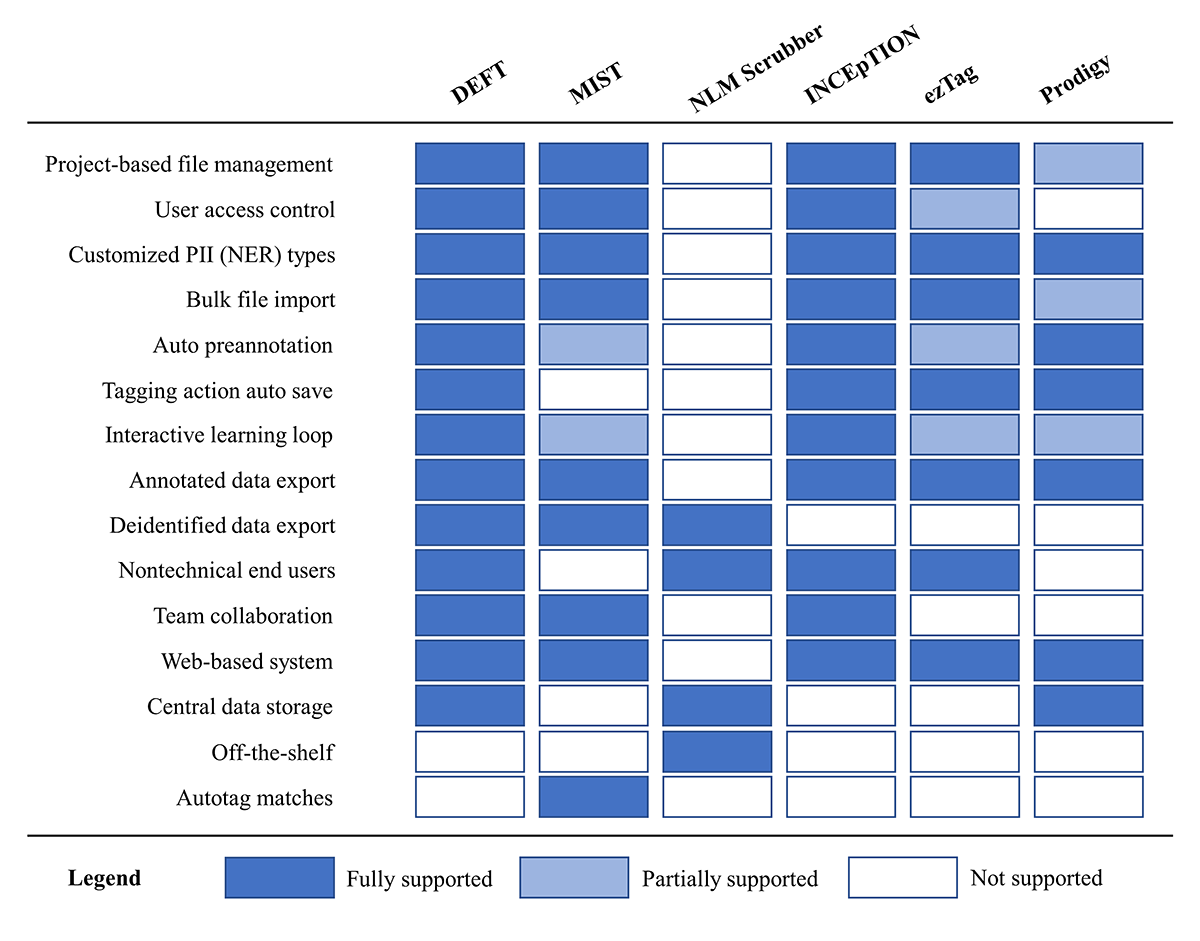
Comparison of the selected tools and DEFT. DEFT: deidentifying free text; MIST: MITRE Identification Scrubber Toolkit; NER: named entity recognition; NLM: National Library of Medicine; PII: personally identifiable information.

We selected 2 deidentification systems (MIST and NLM Scrubber) for functionality comparison with DEFT. We chose these systems because MIST has a similar design strategy of “human in the loop” to DEFT, and NLM Scrubber is an accessible open-source off-the-shelf deidentification system. We also considered INCEpTION, ezTag, and Prodigy, which are text annotation systems with interactive learning loops, because they can be used for the deidentification task with extra effort from technical or ML experts. The system features we compared are (1) support for project-based free text file management; (2) support for user access control; (3) support for customized PII (NER) types; (4) support for bulk file import; (5) support for automated preannotation based on a pretrained model; (6) support for auto save of the tagging actions; (7) support for interactive learning loop; (8) support for annotated data export; (9) support for deidentified data export; (10) suitability for nontechnical end users; (11) support for team collaboration; (12) web-based system; (13) central data storage; (14) off-the-shelf; and (15) autotag matches (all occurrences are automatically tagged when annotators tag a PII entity in the whole working free text). Figure 6 shows that DEFT, MIST, and INCEpTION support the user access control functionality, which is important in deploying the systems for team collaboration on the deidentification task. Otherwise, any devices on the same network can access the system via the system’s URL to potentially access the identified data. Although ezTag provides a session-based login, it is not a secure way to control access because anyone who has the session URL can access the project freely. Both MIST and Prodigy require end users to use a command-line interface to configure the projects or data sets and retrain the models in the interactive learning loop. MIST and ezTag need to manually trigger the model retraining and preannotation from the command-line interface and the web UI, respectively. When starting the Prodigy system, a path to the free-text data needs to be configured. Therefore, it partially supports the project management and bulk file import. Different from other systems, MIST must manually save each tagging action by the user clicking on the save button. In both the INCEpTION and ezTag systems, importing the free-text data requires transferring the files from the original data location to the specified location using the web UI, while MIST uses the command-line interface to do the same thing. This could be a bottleneck when importing large volumes of data sets due to network delays. Because NLM Scrubber is an off-the-shelf desktop software, most of the comparison functionalities are not supported by it. All the systems except NLM Scrubber cannot be used “out-of-the-box.” Only the MIST system provides “Autotag matches” functionality.

Fewer mouse clicks make the annotation process more efficient. We counted the mouse clicks of the add-PII and remove-PII operations for all the selected systems except NLM Scrubber, which is pretrained and doesn’t support annotation. The “drag-and-release” action is counted as 1 mouse click. As shown in Table 2, DEFT and Prodigy had the fewest clicks for both operations. MIST and INCEpTION needed 3 mouse clicks to annotate a PII entity. However, MIST provides an “Autotag matches” functionality, which can automatically tag all the occurrences of the same word spans of the selected PII entity in the whole working file. ezTag needed the most mouse clicks to remove a tagged PII entity.

We evaluated the performance of our model by comparing it with 5 benchmark models [14,17,18,23,24] on the 2014 i2b2 data set. The microaverage strict entity–level scores and binary PII token–level scores are reported in Table 3. Our model achieved the highest strict entity–level recall and *F*_1_-scores at 0.9563 and 0.9627, respectively. Table S1 in Multimedia Appendix 1 lists the hyperparameters used for model training. The microaverage scores by i2b2 category for strict entity matching are shown in Table S2 in Multimedia Appendix 1.

**Table 2:** Mouse click comparison of add-PII^a^ and remove-PII operations.

Operation	DEFT^b^	MIST^c^	NLM^d^ Scrubber	INCEpTION	ezTag	Prodigy
Add-PII^e^	1-2	3	N/A^f^	3	1-2	1-2
Remove-PII	1	2	N/A	2	3	1

^a^PII: personally identifiable information.

^b^DEFT: deidentifying free text.

^c^MIST: MITRE Identification Scrubber Toolkit.

^d^NLM: National Library of Medicine.

^e^The mouse-click number of add-PII operation for DEFT, ezTag, and Prodigy can be 1 or 2, depending on whether the relevant PII type has been selected or not.

^f^N/A: not applicable.

**Table 3:** Microaverage scores comparison on the 2014 i2b2 data set.

Model (reference)	Model architecture	Strict entity-level				Binary PII^a^ token-level		
		Precision	Recall	*F*_1_-score	Precision		Recall	*F*_1_-score
Liu et al [24]	Ensemble model	0.9646	0.938	0.9511	0.993		0.9728	0.9828
Kim et al [23]	Ensemble model	0.9704^b^	0.9445	0.9573	0.9916		0.9806	0.9861
Tang et al [17]	BiLSTM-CRF^c^ (BERT^d^)	0.9599	0.9502	0.955	0.9902		0.9838^b^	0.987^b^
Catelli et al [18]	BiLSTM-CRF	0.9653	0.9506	0.9579	0.991		0.9755	0.9832
Liu et al [14]	Ensemble model	0.969	0.9559	0.9624	0.9943^b^		0.9786	0.9864
Our model	BiLSTM-CRF (RoBERTa)	0.9692	0.9563^b^	0.9627^b^	0.9892		0.9766	0.9829

^a^PII: personally identifiable information.

^b^The best result for each metric.

^c^BiLSTM-CRF: Bidirectional Long Short-Term Memory-Conditional Random Field.

^d^BERT: Bidirectional Encoder Representations from Transformers.

### Use Case

To evaluate its efficiency and performance, we used DEFT to deidentify clinical notes for the project CardiacAI [42]. CardiacAI is a prospective data repository that collects EMR data for patients with cardiac issues who are admitted to a group of participating hospitals in New South Wales, Australia. The EMR data are linked to state-wide hospital and emergency department visits, the state’s death registry, as well as mobile health remote monitoring data. The aim of this data collection is to enable collaborative research, facilitate the use and rapid translation of state-of-the-art tools and technologies, and ultimately drive improvement in patient care and outcomes. The CardiacAI data repository holds data from January 1, 2017 onwards. The repository currently holds EMRs of 44,201 patients with 61,721 individual hospitalizations to a cardiac, cardiothoracic, or vascular surgical specialty. There are 2,087,737 clinical documents including discharge summaries, progress notes, and other semistructured forms with an average of 34 (SD 44) documents per hospitalization. Discharge summaries have undergone some rule-based deidentification where the document header, which contains structured identifier fields, has been removed. However, embedded identifiers remain within the unstructured text.

The DEFT system was deployed on a workspace with 32 GB memory and 8 vCPU in the E-Research Institutional Cloud Architecture [41] environment. A project named “CardiacAI,” and 2 data sets named “Discharge Summary” and “Progress Notes” were created in the system. According to the data content and research requirements, 5 PII types (PERSON, identification number [IDN], DOB, PHONE, ADDRESS) were defined for the project. At the beginning, we randomly selected 400 clinical notes including 200 discharge summaries and 200 progress notes to be manually annotated using the DEFT end user UI by 1 annotator who is an experienced health-domain data analyst. An intermediate NER model was automatically trained by DEFT using the 400 clinical notes with a split ratio of 0.8, 0.1, and 0.1 for training, validation, and test, respectively. Table 4 shows the model performance on the test set. Nearly 81% PII entities were person type and only 2 were address type. The model achieved a micro-*F*_1_-score of 0.9432.

To compare annotation time with and without auto-preannotation, we randomly selected another 200 clinical notes that consisted of 100 discharge summaries and 100 progress notes and split them into 10 rounds, of which each round contained 10 discharge summaries and 10 progress notes. We also considered the text length when splitting the data to make sure that the compared rounds had similar total words as other rounds. The annotator manually tagged the PIIs in the first 5 rounds without auto-preannotation and continued to complete the last 5 rounds with auto-preannotation enabled. Each round was completed in a single session and the annotation time was recorded. As shown in Table 5, the total time of 5 rounds with-preannotation was about 58 minutes, which was approximately 40% less than the total time of 5 rounds without preannotation (~98 minutes). Moreover, the annotation of the last 5 rounds (with preannotation) was much quicker than the one of the first 5 rounds, despite there being more PII entities in the last 5 rounds. Through the 5-round comparison, the preannotation by the trained model sped up the annotation work by 43% (95% CI 33.9-52.1; *P*<.001).

To compare manual annotation performance without and with preannotation, a gold standard corpus was created by having 2 annotators independently review and correct the annotations of the 200 clinical notes. All disagreements were resolved through a consensus meeting between the 2 annotators. The performance results are shown in Table 6. Manual annotation with preannotation by the pretrained model achieved higher overall microaverage recall and *F*_1_-scores by 0.013 and 0.0053, respectively. Although there were a few false negative and positive errors on the PHONE and ADDRESS types, manual annotation with preannotation demonstrated higher accuracy on the PERSON type (the majority type within the data set) than manual annotation without preannotation.

We reconfigured the retraining threshold to 200 to trigger the system to retrain the model using the whole 600 clinical notes. The results are shown in Table 7. Although the test sets were different between this training and the previous training, we do observe that the overall *F*_1_-score increased by 0.0075, compared to the previous intermediate model trained on 400 clinical notes. The new model improved the *F*_1_-score of DOB and PHONE PII types by large margins of 0.1367 and 0.1298, respectively. Moreover, the recall, which is the most important metric for the deidentification tasks, was improved for each PII type, except ADDRESS.

**Table 4:** The NER^a^ model performance on the test set from the corpus with 400 clinical notes.

PII^b^ type	Precision	Recall	micro-*F*_1_-score	PII entity number
PERSON	0.9657	0.9505	0.958	444
IDN^c^	1	0.9649	0.9821	57
DOB^d^	0.875	0.8077	0.84	26
PHONE	0.7273	0.7273	0.7273	22
ADDRESS	0.3333	0.5	0.4	2
Overall	0.9519	0.9347	0.9432	551

^a^NER: named entity recognition.

^b^PII: personally identifiable information.

^c^IDN: identification number.

^d^DOB: date of birth.

**Table 5:** Annotation time comparison.

Without preannotation						With preannotation				
Round	Clinical note number	Annotation time (min)	PII^a^ entity number	Word number	Round		Clinical note number	Annotation time (min)	PII entity number	Word number
1	20	13:03	81	2687	1a		20	6:28	113	2774
2	20	15:56	117	4089	2a		20	7:55	184	4153
3	20	19:08	155	5198	3a		20	9:31	179	5369
4	20	20:24	139	7133	4a		20	12:13	185	7404
5	20	29:05	189	11,503	5a		20	22:08	300	12,304
Total	100	97:36	681	30,610	Total		100	58:15	961	32,004

^a^PII: personally identifiable information.

**Table 6:** Performance comparison without and with preannotation.

Without preannotation					With preannotation			
PII^a^ Type	Precision	Recall	micro-*F*_1_-score	PII Type		Precision	Recall	micro-*F*_1_-score
PERSON	0.9980	0.9675	0.9825	PERSON		0.9983	0.9851	0.9917
IDN^b^	1	1	1	IDN		1	1	1
DOB^c^	1	1	1	DOB		1	1	1
PHONE	1	1	1	PHONE		0.9667	0.9667	0.9667
ADDRESS	1	1	1	ADDRESS		0.8571	1	0.9231
Overall	0.9985^d^	0.9756	0.9869	Overall		0.9958	0.9886^d^	0.9922^d^

^a^PII: personally identifiable information.

^b^IDN: identification number.

^c^DOB: date of birth.

^d^The best result for each metric between performance without and that with preannotation.

**Table 7:** The NER^a^ model performance on the test set from the corpus with 600 clinical notes.

PII^b^ type	Precision	Recall	micro-*F*_1_-score	PII entity number
PERSON	0.9423	0.9583	0.9502	528
IDN^c^	1	0.9762	0.988	84
DOB^d^	0.9844	0.9692	0.9767	65
PHONE	0.8571	0.8571	0.8571	35
ADDRESS	1	0.5	0.6667	6
Overall	0.9487	0.9526	0.9507	718

^a^NER: named entity recognition.

^b^PII: personally identifiable information.

^c^IDN: identification number.

^d^DOB: date of birth.

In order to evaluate the deidentification efficiency, we used the trained model to deidentify CardiacAI clinical notes that summarized the encounters of patients who have had heart failure, containing on average about 170 words. The whole process took about 95 hours to export 280,785 deidentified clinical notes (about 0.82 seconds per clinical note). There are on average about 5 PII entities per clinical note.

## Discussion

### Principal Findings

We have presented the design and implementation of DEFT, a simple web-based deidentification system with interactive learning loop. DEFT provides a task-focused web UI to end users so that annotation of PII can be done quickly and easily. In addition, an admin web UI is provided to manage the projects, users, and team collaboration on the deidentification task. The DEFT system can be deployed on a central server to provide deidentification services to multiple teams within the same network environment at the same time through user access control functionality.

The results of the functionality comparison showed that our DEFT system is better than the selected comparison systems on the deidentification task, in terms of the ability to customize the task, ease and security of management of users and files, automation of the interactive learning loop, and central data storage.

DEFT can easily customize PII types at the project level using the admin web UI. It provides flexibility for determining the level of deidentification according to risk assessment and analysis requirements of the project. The selected systems which we compared with DEFT have similar customization functionality, with the exception of NLM Scrubber. However, only INCEpTION and ezTag provide a web UI for this function. MIST and Prodigy uses either a configuration file or command line to predefine PII types. NLM Scrubber uses fixed HIPAA-compliant PII types, which restrict the adoption of the system outside of a HIPAA-covered country or organization [6].

DEFT has a 2-level file management structure (ie, project and data set) which allows the users to organize the data more flexibly, compared to the 1-level structure (ie, project) of the other systems. For example, the deidentification project may have many different types of clinical notes. Users can create different data sets for each type of clinical note. Like INCEpTION, DEFT can easily manage user credentials and assign user access to the project level through the admin web UI. However, in the MIST system, user management and access control need to be done from a command-line interface, and all the users share 1 project access key. When starting up the Prodigy system, the users need to configure an input data source. Therefore, the different project users cannot work on the system at the same time. Moreover, there is no user access control functionality in Prodigy. ezTag uses a session-based login to generate a unique URL for each user to manage the user access. However, the session can be accessed by anyone with the unique URL, so it is not suitable for annotating sensitive information in EMR free-text data.

DEFT supports a fully automated interactive learning loop. The model training process is triggered when the newly annotated files reach the preset retraining threshold. The retrained model is automatically loaded for the relevant project. When the annotator opens a nonannotated file, the model automatically preannotates it to provide suggestions for the annotator. In contrast, MIST, Prodigy, and ezTag need human manual operations during the learning loop. MIST retrains a model and preannotates the free-text files via a command-line interface. Moreover, the system needs to be restarted to load the retrained model. Similarly, Prodigy also needs different command lines to perform retraining and preannotation operations. Users of ezTag need to click the “Auto Annotation” and “Train” buttons to trigger the relevant tasks. The real-world use case study we present demonstrated that DEFT’s annotation speed can be increased by 43% with the automated preannotation in the learning loop. Furthermore, the DEFT system can use only 600 annotated clinical notes to achieve good performance with an *F*_1_-score of 0.9507, which is greater than 0.95, the rule-of-thumb benchmark for evaluating the reliability of a deidentification system [16,40]. The low scores for ADDRESS PII entity as shown in Tables 4 and 7 may be caused by the lack of training samples, as there were only 44 and 55 ADDRESS entities in the training sets of 400 and 600 clinical notes, respectively. The model performance will continually improve as the amount of annotated free-text data increases. We also evaluated the annotation time and accuracy of manual annotation without and with preannotation. Using preannotation during the manual annotation process resulted in a time savings of 43% and a micro-*F*_1_-score improvement of 0.005 compared to manual annotation performed without preannotation.

The design of DEFT allows the raw free-text data to be stored in a central data storage location which is under the control of the researchers or data custodians. The system-related data, such as project and data set information, users, PII index and type details, and the free-text file names, are saved in DEFT’s database. All the raw data and exported data are managed centrally by the data manager. This not only protects the identified data from unauthorized access, but also avoids transferring large volumes of data through the network. A data source path is required when starting up the Prodigy system and therefore data can be stored at a central location. However, none of the other systems support this.

In the traditional annotation process, more than 2 annotators are needed to generate a gold standard data set. The interannotator agreement is measured to evaluate the annotation reliability between different annotators. Although a single annotator was used in this case study, the interactive learning loop enabled the model and the annotator to suggest and correct each other to continually improve the model performance. However, DEFT can easily perform the traditional annotation process. For example, the data manager creates a data set named “gold standard” in the project. In total, 2 annotators are required to tag the PIIs from all the files in the data set. Another annotator as an audit changes the file status to completion after reviewing the annotation results. The NER model will be trained on the gold-standard data set.

### Limitations

In DEFT, we preannotate all the PII from the free text using the trained NER model. The annotators need to review the whole text to correct the preannotations. In contrast, INCEpTION and Prodigy combine preannotation with active learning which queries the user for feedback (accept, reject, or skip) on the annotation suggestions that are most informative to the model [30]. Active learning can achieve rapid and accurate annotation with less annotation time [43]. Integration of active learning within the learning loop could enhance the next version of DEFT. Moreover, we will explore fine-tuning pretrained transformer language models for NER in DEFT to improve the accuracy of deidentification. Furthermore, implementing “Autotag matches” in DEFT could have the potential to further decrease manual effort involved in the annotation process. Another limitation of DEFT is that it relies solely on deep learning models. The system’s performance could potentially be enhanced by incorporating a hybrid method that combines knowledge-based methods (such as predefined regular expressions or knowledge dictionaries) and deep learning. The evaluation results in this study are derived from clinical notes which were annotated by a single annotator. This may introduce potential reliability issues when assessing the deidentification performance. To address this, an annotation process, which is manually annotated by 3 annotators (2 annotators and 1 adjudicator), is needed to be introduced in the next version of DEFT.

### Conclusions

DEFT is a web-based deidentification system, which is designed for health domain researchers and data custodians to easily deidentify free-text data in EMRs with the support of an interactive learning loop. End users can perform all the operations through a well-designed web UI. DEFT has many good features to help manage and organize the deidentification tasks. In particular, the central data storage feature ensures that the identified data are protected properly in a central location without transfer through the network. The real-world use case demonstrated that DEFT can speed up the annotation process and quickly complete the deidentification work for large volumes of data with high reliability. The source code of the DEFT system is available at GitHub [44].

## Appendix

Multimedia Appendix 1 Supplemetary material.

## Abbreviations

BiLSTM-CRF: Bidirectional Long Short-Term Memory-Conditional Random Field
DEFT: deidentifying free text
DOB: date of birth
EMR: electronic medical record
HIPAA: Health Insurance Portability and Accountability Act
IDN: identification number
MIST: MITRE Identification Scrubber Toolkit
ML: machine learning
NER: named entity recognition
NLM: National Library of Medicine
PII: personally identifiable information
UI: user interface

## Data Availability

The public 2014 i2b2 data set repository can be accessed with registration on the website of Department of Biomedical Informatics at Harvard Medical School [45]. Access to CardiacAI data repository must be assessed and approved by the CardiacAI Data Governance Committee. Please visit the CardiacAI website [46] for more information about eligible research proposals.
