# Supplementary material for: Web-Based Application Based on Human-in-the-Loop Deep Learning for Deidentifying Free-Text Data in Electronic Medical Records: Development and Usability Study
Source: Interact J Med Res. 2023 Aug 25;12:e46322. doi: 10.2196/46322 (PMC10492176; doi:10.2196/46322)
Supplement: Multimedia Appendix 1 [file ijmr_v12i1e46322_app1.docx]

**Figure S1.** An example of a dummy annotated discharge summary in the XML format. Each tag item contains start and end positions in the original text, PII entity content, PII entity type, and annotator name.


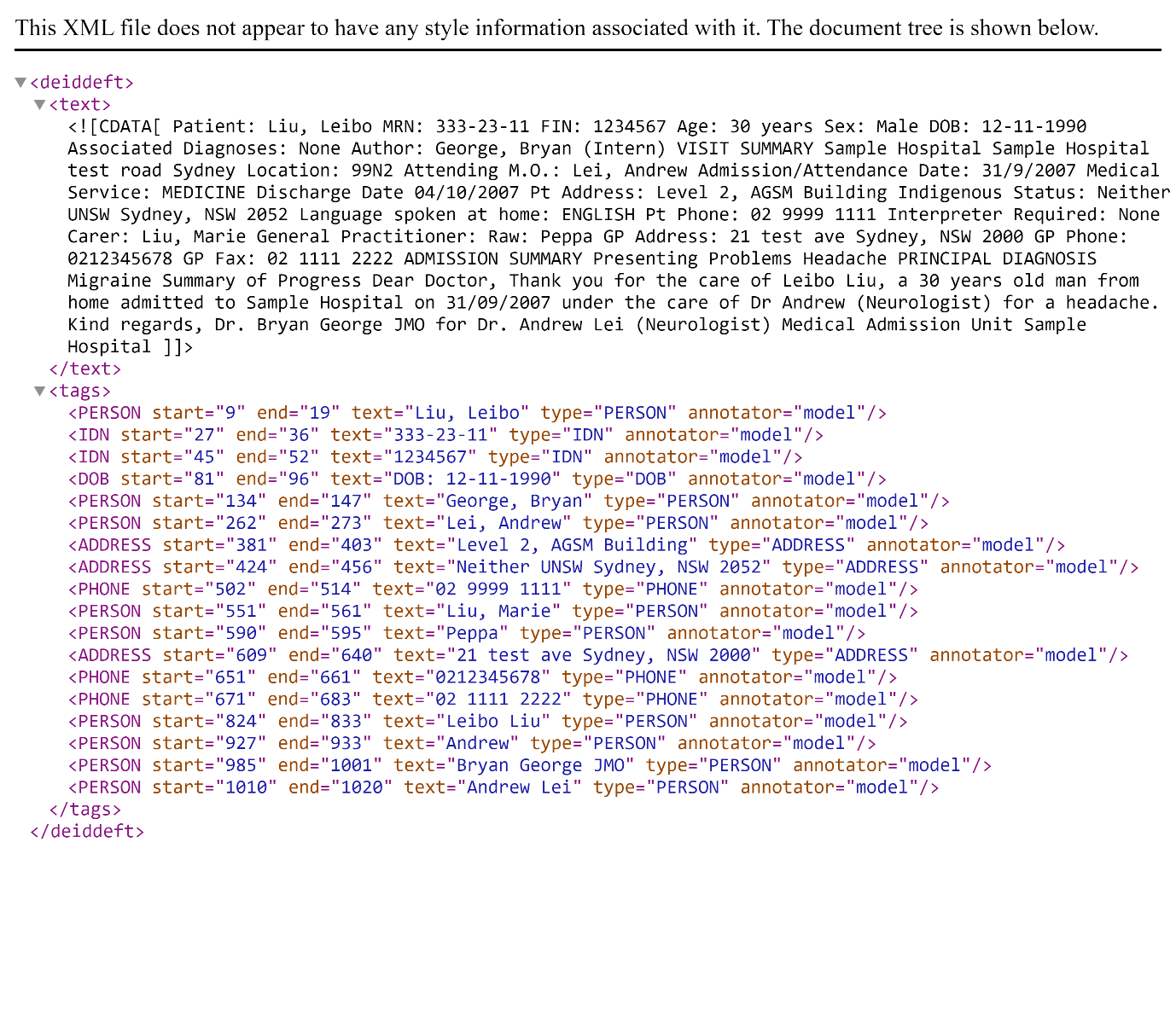


**Figure S2.** An example of a dummy deidentified discharge summary in the txt format.


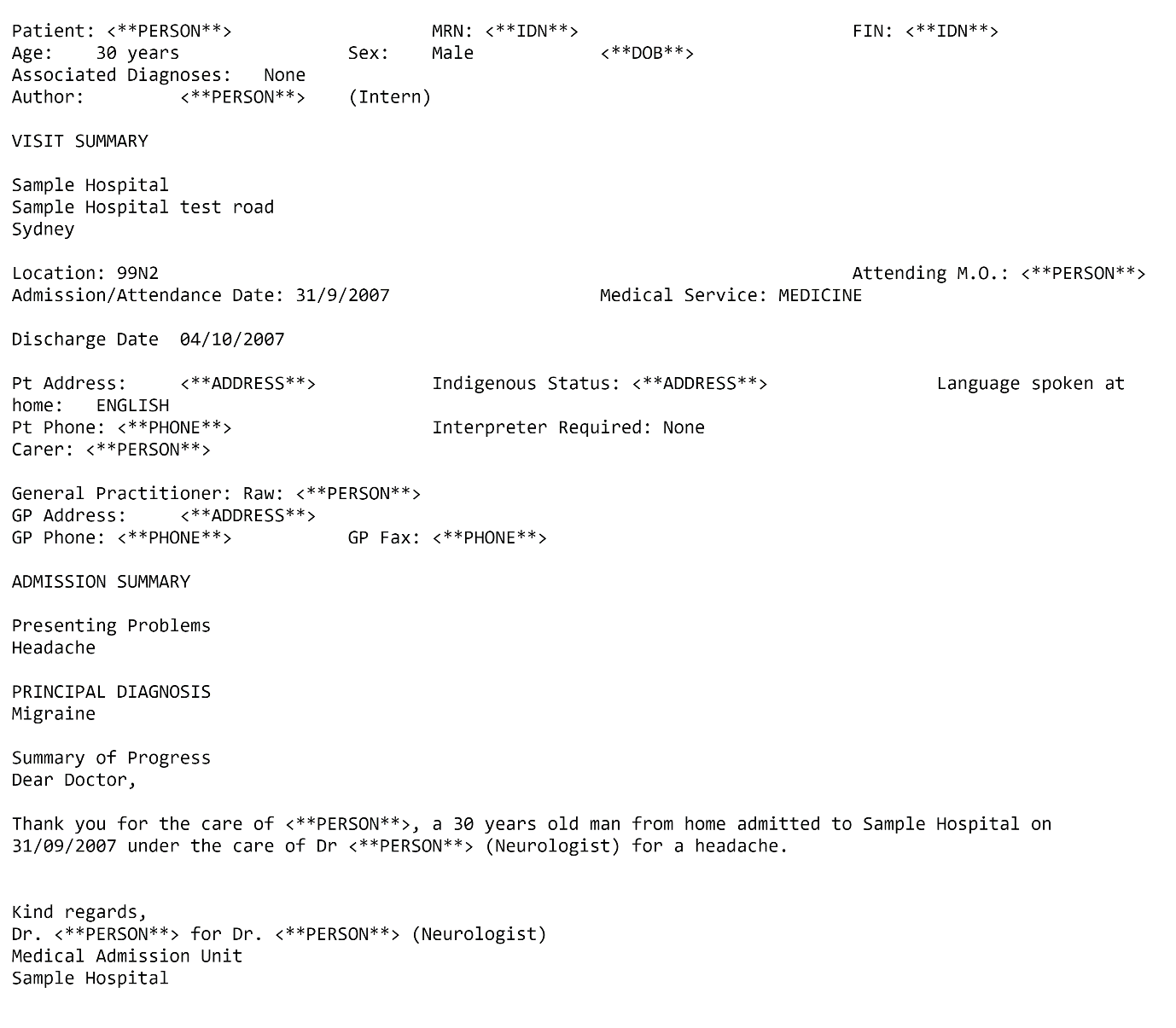


**Table S1.** Hyperparameters used for model training on the 2014 i2b2 data set.

| Hyperparameter | Value |
| --- | --- |
| Learning rate | 0.1 |
| Batch size | 32 |
| Dropout | 0.5 |
| Max epochs | 100 |
| Word Embedding | RoBERTa-base-PM-M3-Voc-distill-align^[[1]](#footnote-1)^ |

**Tables S2.** Microaverage strict entity-level scores by i2b2 category on the test set of the 2014 i2b2 data set.

| **i2b2 Category** | **Strict entity-level** | | |
| --- | --- | --- | --- |
|  | Precision | Recall | F1 |
| AGE | 98.68 | 98.29 | 98.49 |
| CONTACT | 89.43 | 93.98 | 91.65 |
| DATE | 99.01 | 98.53 | 98.77 |
| ID | 95.58 | 93.44 | 94.50 |
| LOCATION | 92.72 | 87.29 | 89.92 |
| NAME | 96.87 | 96.53 | 96.70 |
| PROFESSION | 86.39 | 83.43 | 84.88 |
| Total | 96.92 | 95.63 | 96.27 |

1. https://github.com/facebookresearch/bio-lm [↑](#footnote-ref-1)
